# Immunopsychiatry – Innovative Technology to Characterize Disease Activity in Autoantibody-Associated Psychiatric Diseases

**DOI:** 10.3389/fimmu.2022.867229

**Published:** 2022-05-23

**Authors:** Niels Hansen

**Affiliations:** ^1^Department of Psychiatry and Psychotherapy, University Medical Center Göttingen, Göttingen, Germany; ^2^Translational Psychoneuroscience, University Medical Center Göttingen, Göttingen, Germany

**Keywords:** immunopsychiatry, anti-neural autoantibody, innovative technology, methods, autoimmunity

## Abstract

**Background:**

Anti-neural autoantibody-associated psychiatric disease is a novel field in immunopsychiatry that has been attracting attention thanks to its potentially positive therapeutic outcome and distinct prognosis compared with non-organic psychiatric disease. This review aims to describe recent novel technological developments for improving diagnostics in the field of autoantibody-related psychiatric disease.

**Methods:**

We screened for relevant articles in PubMed for this narrative article. We focused on research methods such as neuroimaging, immune cells and inflammation markers, and molecular biomarkers in human biofluids like serum and cerebrospinal fluid and plasma proteomics.

**Results:**

We introduce several novel methods for investigating autoinflammation with the aim of optimizing therapies for autoantibody-associated psychiatric disease. We describe measuring the translocator protein 18kDa in activated microglia via positron emission tomography imaging, brain volumetric assessment, flow cell cytometry of cerebrospinal fluid and blood, and blood biological probes as well as psychopathological cues to help us gain insights into diagnosing inflammation and brain damage better in psychiatric patients presenting a suspected autoimmune etiology.

**Conclusion:**

Our short methodological review provides an overview of recent developments in the field of autoantibody-related immunopsychiatry. More research is needed to prove their usefulness in diagnosing and treating autoantibody-associated psychiatric disease and its subtypes.

## Introduction

Immunopsychiatry is a novel and expanding subfield of psychoneuroimmunology that deals with the interaction between our immune system and psychiatric disorders. Neural autoantibodies in psychiatric disease are attracting more attention due to their broad spectrum and link to disease pathogenesis. Autoantibody-associated psychiatric disorders are of growing interest as the therapy is demanding and differs from standard psychopharmacological drug therapy. Psychiatric disorders associated with neural autoantibodies can be classified as autoimmune-mediated psychiatric disease when these neural autoantibodies occur in conjunction with specific clinical features, signs of central nervous system inflammation in neuroimaging, or in biomaterial probes such as cerebrospinal fluid (CSF) (1–4).

## Antineural Autoantibodies in Psychiatric Disease – Novel Developments

A still enigmatic topic in autoantibody-related immunopsychiatry is whether psychopathology is influenced or even driven by the presence of autoantibodies. A recent study (5) showed that specific psychopathologic features are associated with specific autoantibodies, thus highlighting the relationship between a psychopathology’s pathogenesis and autoantibodies. A large sample of 461 psychotic patients was screened for autoantibodies in this study. Psychosis is a severe and debilitating disease condition characterized by psychopathology consisting of delusions, hallucinations or ego disturbances. They screened a subcohort of 24 patients for autoantibodies *via* protein arrays and detected six different autoantibodies associated with specific psychopathological features. Anti-adaptor-related protein complex 3, beta 2 subunit (AP3B2) antibodies were identified in conjunction with persecutory delusions, whereas anti-tryptophan 2,3-dioxygenase (TDO2) antibodies were more frequently co-detected with hallucinations in patients. Affective symptoms such as anhedonia and dysphoria were related to anti-recombinant WAS protein homologously associated with actin, Golgi membranes and microtubules pseudogene 3 (WHAMMP3) antibodies. Cognitive dysfunction was found to be mainly associated with anti-olfactomedin 1 (OLFM1) antibodies, and sleep dysfunction (insomnia) in patients was associated with anti-Gamma-crystallin N (CRYGN) antibodies. These examples in Jernbom Falk´s study (2021) demonstrate that specific psychopathologic features might help deliver clues about which autoantibodies might be worth searching for. However, the likelihood of detecting autoantibodies is much higher if specific syndromes are present, ie, a psycho-organic syndrome accompanying cognitive dysfunction or paranoid-hallucinatory syndrome, as a recent cohort study showed that psychotic symptoms and cognitive impairment are the most relevant psychiatric conditions associated with autoantibodies (6). Although the occurrence of autoantibodies in first-episode psychosis is rare (7), neural autoantibodies are detected in first-episode psychosis, thus requiring a differential diagnosis including the search for autoantibodies. However, it is unclear which kind of first-episode psychotic patients warrant an autoantibody search. Unspecific indicators like autoimmune indicators are relevant, such as an actual or recent tumor diagnosis, movement disorder, altered consciousness, seizures, optic hallucinations, infectious prodrome with fever or aphasia, dysarthria and mutism (1, 3, 8). We do not yet know how these indicators are weighted specifically, apart from grouping them into minor and major indicators. According to our recent review (1), major indicators like seizures or a decreased level of consciousness and minor autoimmune indicators such as early resistance to therapy and fluctuating psychopathology can be distinguished. There would be major practical consequences if we knew which specific psychopathological parameters indicate a potential anti-neural autoantibody-mediated psychosis, for instance. In light of these reflections, Jernbom Falk´s study findings (2021) are very promising. We tested the psychopathological profiles of a cohort of psychiatric patients and found that psychiatric syndromes that are mainly psycho-organic and depressive reveal fewer affective symptoms in specific categories (like affective rigidity or rumination) than those patients without anti-neural autoantibodies (unpublished observations). These findings indicate that patients with a psycho-organic and depressive syndrome associated with neural-autoantibodies might have a psychopathology different from that in patients not presenting (anti-neural autoantibodies). This factor indicates the direction of potential psychopathological patterns associated with psychiatric disease in conjunction with autoantibodies. These studies illustrate the relevance of psychopathology for diagnostic purposes apart from grouping patients into diagnostic classification systems such as international classifications of diseases.

## Potential Pathogenesis of Autoantibody-Associated Psychiatric Disease

The pathogenesis of neural autoantibody-associated psychiatric disease is still not well understood, although there are pathophysiological models for several autoantibodies implying that autoantibodies per se might play a pathogenic role in the development of psychiatric symptoms and behavior. An animal model recently showed that a neuropsychiatric phenotype of N-methyl-D-aspartate receptor (NMDAR) encephalitis is modulated by NMDAR autoantibodies (9). NMDAR is a glutamatergic receptor responsible for excitatory neuronal plasticity in the brain on the one hand and may trigger excitotoxicity on the other hand in the brain. However, it is debatable whether these autoantibodies cause the encephalitis itself or only modify it, as depicted in a recent study (10). Other studies in mice have shown that it is not the brain’s acute exposure to NMDAR autoantibodies but rather their long-lasting presence that causes a behavioral phenotype entailing impaired cognitive function (11). NMDAR autoantibody-induced alterations in synaptic transmission are believed to be a key step in generating psychotic symptoms concurring with schizophrenia’s glutamatergic model (12). NMDAR autoantibodies lead to NMDAR internalization and removal from the cell surface linking them to an extrasynaptic compartment. The conditions necessary for normal synaptic transmission are thus disturbed. Dysfunctional synaptic transmission could lead to psychiatric problems like psychotic symptoms. NMDAR trafficking to extrasynaptic compartments has been observed in patients with psychotic disorders, but not in autistic patients or healthy controls, as Wollmuth et al. (13) described. Their finding makes it tempting to wonder if there might not be a variety of unknown molecular mechanisms associated with NMDAR antibodies among diverse psychiatric disorders. More research is needed to determine the molecular mechanism induced by autoantibody blockage of NMDAR. Generated monoclonal NMDAR antibodies recently applied to neuronal cultures revealed that these have an impact on disinhibitory signaling in cortical neuronal networks (14). Disturbed inhibitory cortical neurotransmission supposedly results in a hyperexcitable state that could enhance the induction and maintenance of psychotic symptoms. Furthermore, the autoantibody class seems to play a major role in the underlying pathophysiological mechanism. As membrane-surface autoantibodies like NMDAR autoantibodies are probably pathogenic themselves (15), T-cell dependent proinflammatory states are probably irrelevant in relation to a membrane-surface autoantibody-associated neuropsychiatric disease such as NMDAR encephalitis (16), but T-cells are relevant immune cells involved in autoantibodies against intracellular targets associated with neuropsychiatric disease (17). Other novel autoantibodies have been identified playing a potential tole in specific psychiatric diseases, ie, anti-hypothalamus autoantibodies in anorexia nervosa (18). They suggest that these autoantibodies disturb the physiological processes by interacting with hypothalamic cells leading to augmented secretion of anorexigenic signaling (18). Another autoantibody category attracting considerable attention recently is anti-basal ganglia autoantibodies, believed to be pathogenically relevant for a subtype of obsessive compulsive disorder (2, 19). As the basal ganglia play a key role in movement execution, anti-basal ganglia antibodies could play a pathogenic role in interfering with movement causing the repetitive behaviors and actions associated with obsessive compulsive disorder. These examples show how more animal models are needed to investigate specific subclasses of neural autoantibodies in psychiatric disease.

## Novel Discoveries in Methods Relevant for Immunopsychiatry

### Immune Cells in Biological Probes

Immune cells are potential biomarkers in patients with anti-neural autoantibody-related cognitive impairment and epilepsy, as recent investigations of ours showed (1, 20, 21). Recent evidence shows that cell flow cytometry is a promising method to evaluate immune cell subsets in psychotic disorders (22). CSF cell flowmetry is interesting as it helped detect a relative shift in immune cells from lymphocytes to monocytes in psychotic disorders, potentially indicating an altered adaptive immune system. Increased protein concentrations and blood-brain barrier disturbances have also been detected in these patients in line with those findings (22) indicating a leaky blood-brain barrier enabling immune-cell infiltration within the brain in patients suffering from psychotic disorders. These results show that flow cell cytometry is a potentially rewarding tool for clarifying questions related to cellular inflammation in patients with autoantibody-associated psychotic and neurocognitive syndromes. Flow cytometry should be applied with special focus on classic monocytes in psychosis (22) and CD4+ T-cells in verbal memory and executive functions should also be the spotlight of investigation (21). There is very recent and fascinating evidence of higher levels of CD3+CD4+CD25+Fox3 cells (also called regulatory T-cells) in the blood of patients with attention deficit hyperactive disorder compared to healthy controls (23). Elevated fractions of these regulatory T-cells thus seem to raise the risk of attention deficit hyperactive disorder (23). Another study by Gao et al. (24) addressed the usage of flow cell cytometry in monocytes and CD4+ T-cells in the blood of patients with bipolar disorder to measure lithium’s treatment efficacy. Indeed, a special flow- cytometric approach was employed to measure proteins and immune cells in this study. In this study we demonstrate that several proteins such as leptin, brain-derived growth factor, neurotrophin and epidermal growth factor receptor pathways are altered in patients responding to lithium, unlike in those who do not respond to it. Our preliminary findings reveal that flow cytometry in monocytes and CD4+ T-cells is a potentially useful technique when examining biomaterial samples to seek evidence in immunopsychiatry. To investigate a broader spectrum of proteins, other technologies like proteomics are preferable to flow cytometry for measuring proteins; they can supplement flow cytometry.

### Imaging Neuroinflammation

Recent studies have described the potential of measuring neuroinflammation, ie, in schizophrenia (25) as a disease that severely affects cognition, emotions and daily life abilities or depression (26) accompanied by strong mood and psychomotor disturbances (by measuring the translocator protein 18kDa in activated microglia *via* PET imaging (TSPO). TSPO imaging has not yet been adopted in the clinical routine although the evidence regarding the detection of glial neuroinflammation in psychiatric disease has been promising. Further research should be extended to study the interaction between neurotransmitters such as noradrenaline and acethylcholine and neuroinflammation *via* TSPO imaging in psychiatric and age-related, disease-associated anti-neural antibodies. TSPO imaging has proven useful in neuropsychiatric disease associated with autoantibodies such as lupus erythematosus, as (27) demonstrated in a recent study *via* the specific radioligand for TSPO named DPA (^11^C]DPA-713). Stronger DPA binding was shown in hippocampal and cerebellar regions in cognitively-impaired patients with neuropsychiatric lupus erythematosus (27) indicating elevated glia cell activation in these regions. Glia cell activation underlie neuroinflammatory processes in lupus erythematosus patients, thereby yielding specific insights into the disease mechanisms of autoantibody-related psychiatric disease. TSPO imaging is known to facilitate the depiction of neuroinflammation in patients with major depressive disorder, obsessive compulsive disorder (28), and dementia such as Alzheimer´s disease (29).

### Brain Morphology and Inflammation

Further research should be done to discover promising regions to study psychopathologies and how they correlate with brain morphology. A recent study showed that a larger anterior insula volume is related to anxiety severity caused by inflammation (30). Another study (31) confirmed the link between inflammation by measuring cytokines and assessing brain morphology in psychiatric patients. Those authors (31) used blood cytokine markers from antipsychotic-naïve, first-episode psychotic patients, clustering these cytokines in inflammatory subtypes. Theoretical graph analyses were done to connect brain morphology and cytokine patterns classified as inflammatory subtypes in first-episode patients, which facilitated assessments of local and global network characteristics across inflammatory subtypes. Their study demonstrated enhanced regional volume of the right parahippocampal region, caudal anterior cingulate and bank superior sulcus in inflammation subtypes of schizophrenia. A similar link between neuroinflammation and brain volume was confirmed in another recent study with first-episode psychotic patients presenting a proinflammatory profile and elevated interferon gamma and interleukin 5, which were associated with lower total cortical volume (32). Lower cortical volumes might thus be associated with elevated cytokines indicating brain inflammation. Whether the brain inflammation is accompanied by a process involving a loss of brain volume, or the reduced brain volume itself triggers further inflammatory processes is unknown, and deserves further investigation. Another unexplored issue is where in the brain the inflammation in autoantibody-associated psychiatric disease occurs. In a recent study of autoantibody-associated psychiatric disease, inflammatory processes were detected in motor structures like the basal ganglia, and in limbic structures such as the amygdala and hippocampus region (33). However, the relationship between brain morphology and inflammation in autoantibody-associated psychiatric disease is sometimes hard to detect as the inflammation may only be mild and local and thus not visible on MRI or FDG PET neuroimaging exams or evident in CSF analysis. Zrzavy etal. (33) detected neuroinflammation in conjunction with inflammatory infiltration by parenchymal CD3/CD8+ T-cells and CD79a+ B-cells in parts of the basal ganglia, amygdala and hippocampus *via* postmortem immunopathological examinations. We therefore need innovative new methods to detect mild inflammation, as the current standard diagnostic procedures are inadequate. Peripheral inflammation might be informative as additional information for deciphering CNS inflammation, but often fail to correlate with CSF inflammation, as a recent study showed in patients with a schizophrenia-spectrum disorder (34). However, whether peripheral inflammation is useful is controversial, as another study showed upregulated complement mRNA expression in blood samples linked to cortical thinning in schizophrenia (35). More research is needed to clarify the role of peripheral inflammation in brain processes in anti-neural autoantibody-associated psychiatric disease and to take small changes in brain volume into account. Choroid plexus enlargement is another candidate for examination as altered brain structures may indicate an interrelationship between inflammation and brain morphology in term of substructures. A recent study reported an enlarged choroid plexus in patients with depression (36): the choroid-plexus volume correlated with binding in [11C]PK11195 PET/structural MRI imaging (36), confirming the link between neuroinflammation and choroid-plexus morphology. Assessing choroid-plexus morphology in autoantibody-associated psychiatric disease might therefore be a future method to examine inflammation processes that are not yet clearly affecting white or gray matter morphology, but affect the choroid-plexus morphology early.

## Limitations

Anti-neural autoantibody-associated psychiatric disease is a novel field that presents several challenges, namely too little knowledge and too few studies investigating this disease, its underlying pathomechanisms are unknown, and treatment options are rare and have usually not been examined in large patient cohorts. Our understanding of the pathogenesis of autoantibodies often relies on animal models that preclude a direct transfer of evidence to humans. These aspects should be kept in mind when combining autoantibody analysis in novel diagnostic approaches with knowledge about autoantibodies’ pathophysiologic actions. However, only by addressing these questions while applying novel and highly sophisticated techniques can autoantibody-associated psychiatric disease and its many unknown facets be further explored. In addition, we need to study other patterns of brain damage or brain inflammation between psychiatric patients presenting autoantibodies in comparison to psychiatric patients presenting no autoantibodies (apart from specific neural autoantibodies) *via* blood proteomics to identify specific patterns by which to distinguish disease groups.

## Synopsis – Marking Disease Activity in Neural Autoantibody-Mediated Psychiatric Disease

**Figure 1:**
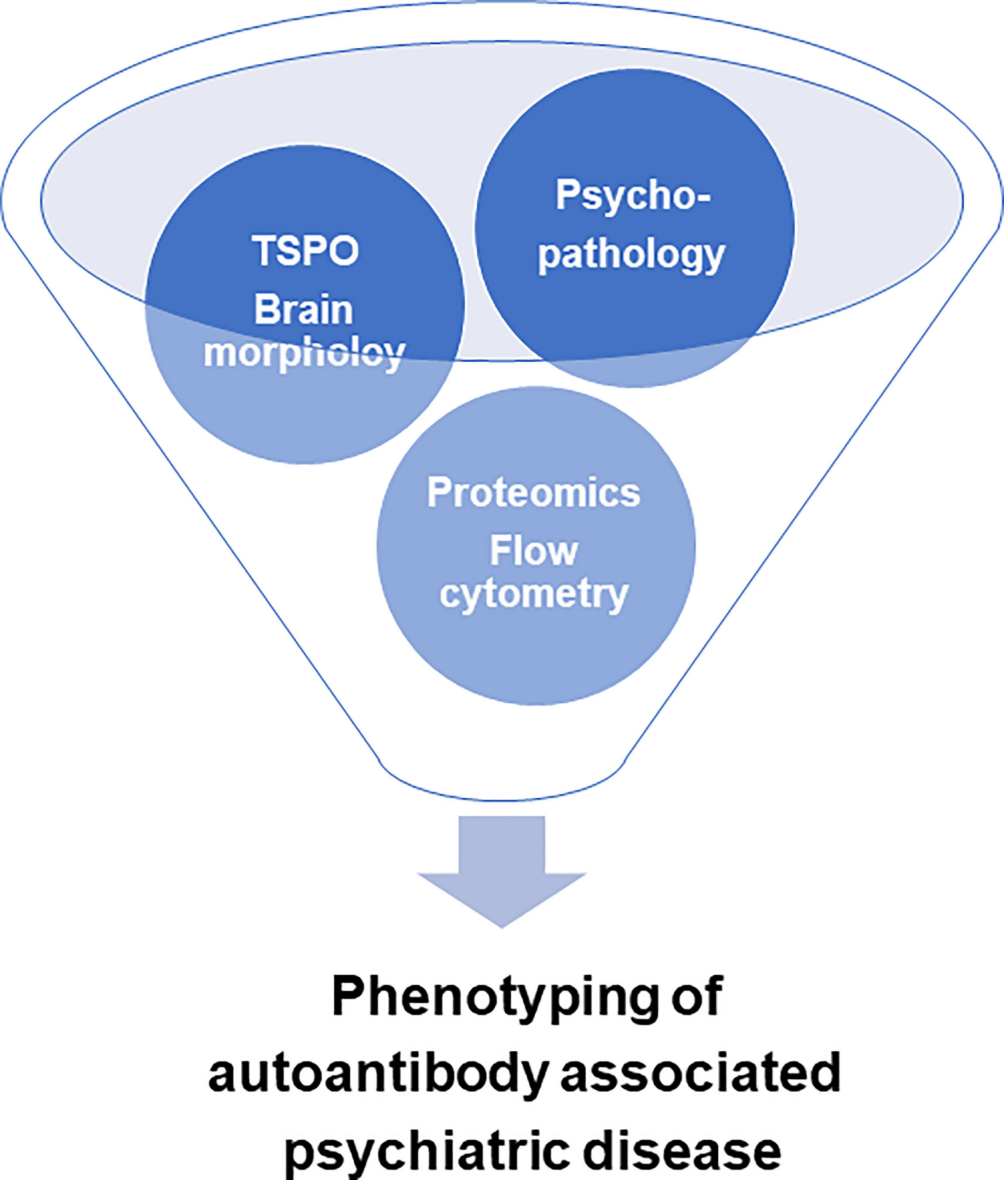
Innovative technology for optimizing the characterization of autoantibody-associated psychiatric disease. TSPO = activated microglia translocator protein 18kDa through positron emission tomography imaging.

Our short review indicates that there are several innovative techniques (summarized in **Figure 1**) to combine to better identify disease activity in psychiatric diseases associated with anti-neural autoantibodies. More effort should be invested to encourage attempts to apply these methods in order to understand the role of anti-neural autoantibodies in psychiatric disease. Different classification criteria have been developed for subtypes of autoantibody-mediated psychiatric disease such as autoimmune psychosis (3), autoimmune obsessive-compulsive disorder (2), and autoimmune dementia (37). More effort is needed to improve and test the diagnostic usefulness of several methods and evidence that have not yet been confirmed in large-scale studies. The methods described in this review also require substantial testing in large populations to prove their diagnostic validity in different psychiatric patient samples in a cross-sectional, but also longitudinal manner.

## Author Contributions

The author confirms being the sole contributor of this work and has approved it for publication.

## Funding

Funding is derived from the Open access fund of the University of Göttingen.

## Conflict of Interest

The author declares that the research was conducted in the absence of any commercial or financial relationships that could be construed as a potential conflict of interest.

## Publisher’s Note

All claims expressed in this article are solely those of the authors and do not necessarily represent those of their affiliated organizations, or those of the publisher, the editors and the reviewers. Any product that may be evaluated in this article, or claim that may be made by its manufacturer, is not guaranteed or endorsed by the publisher.
